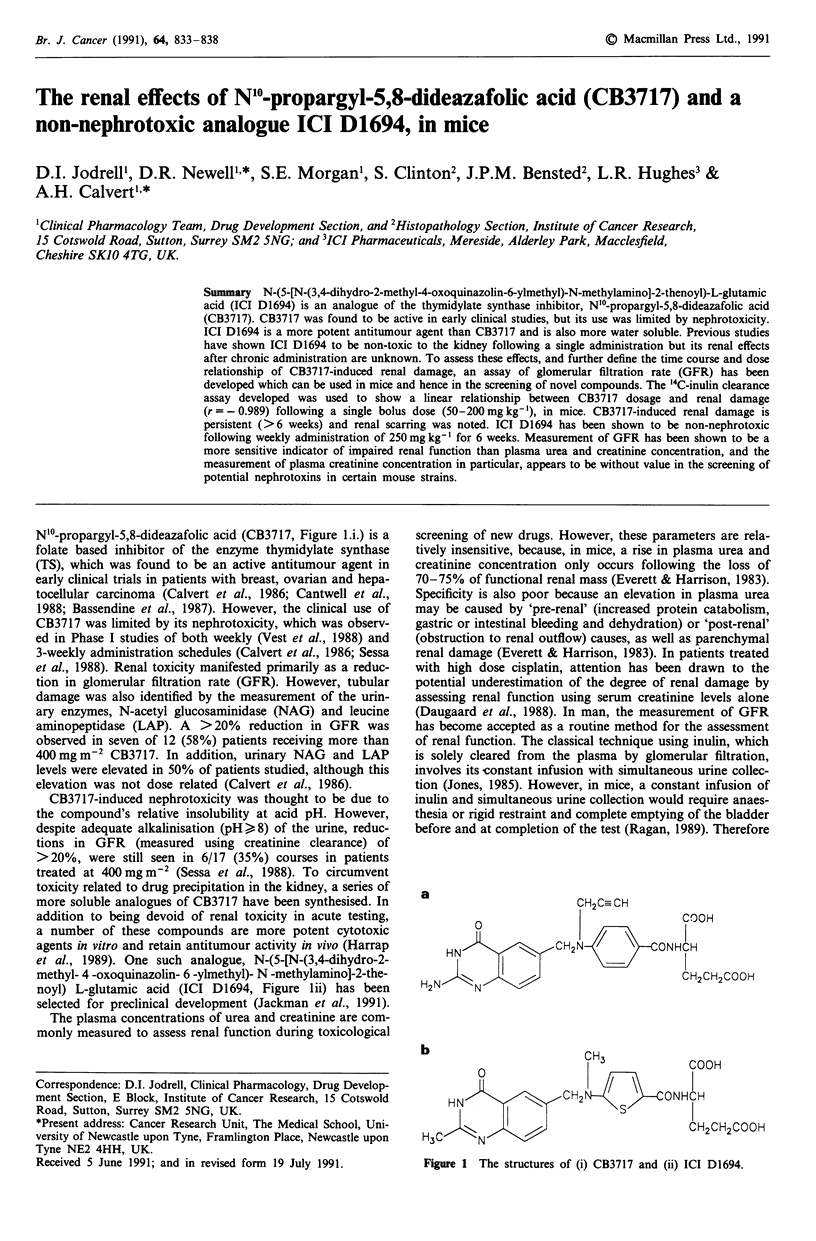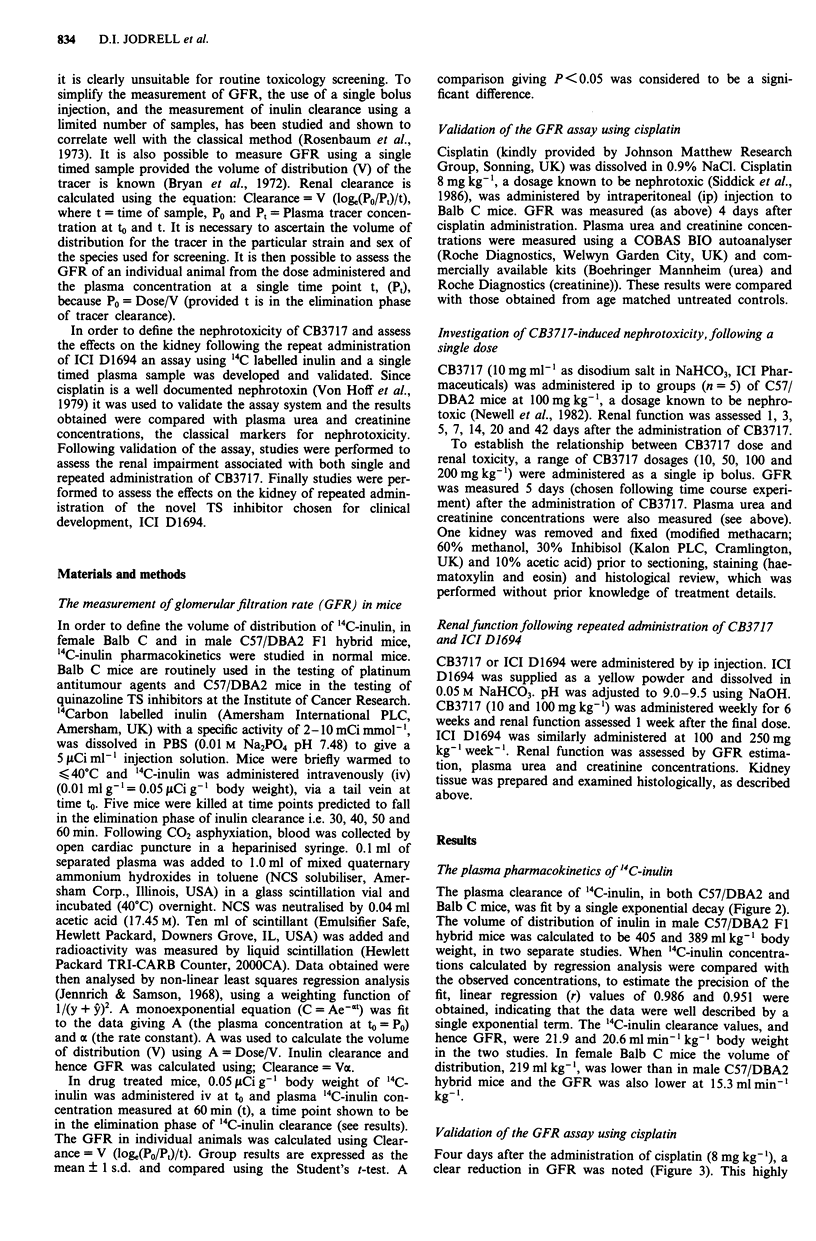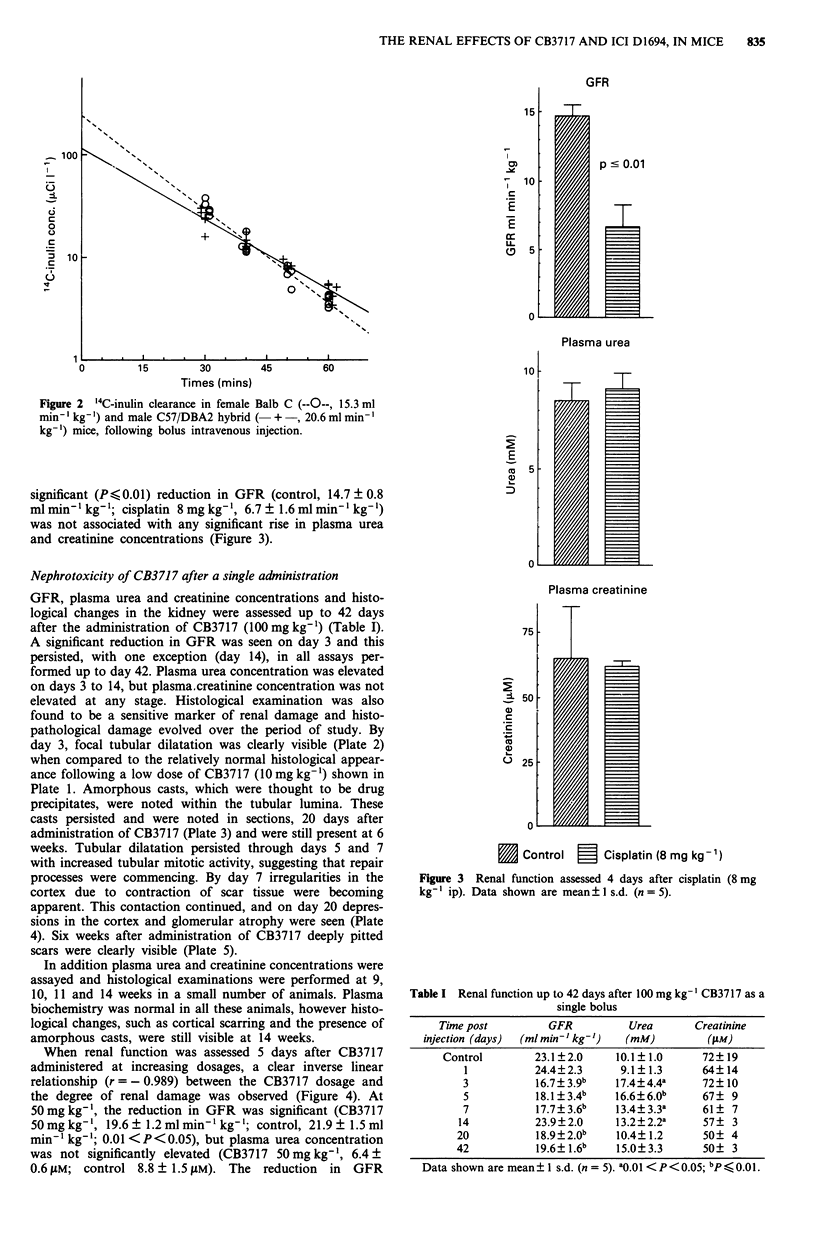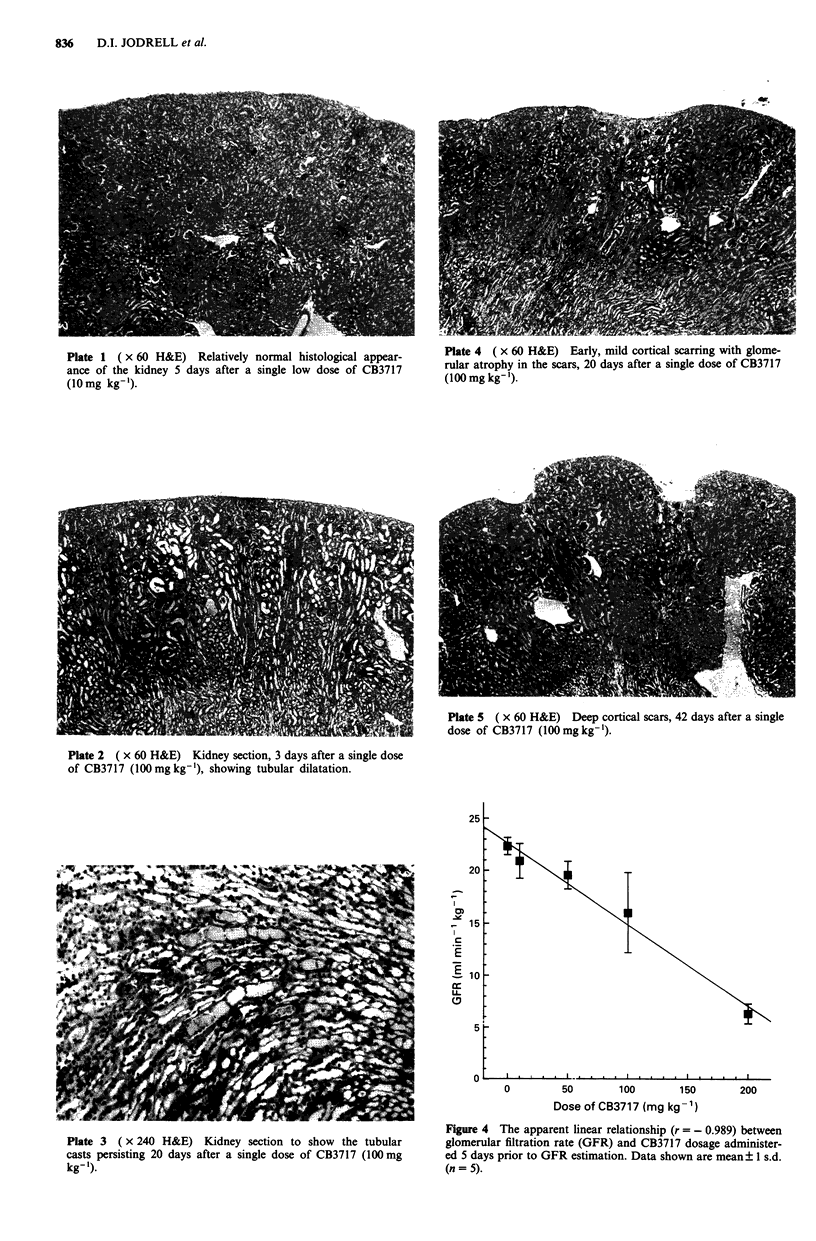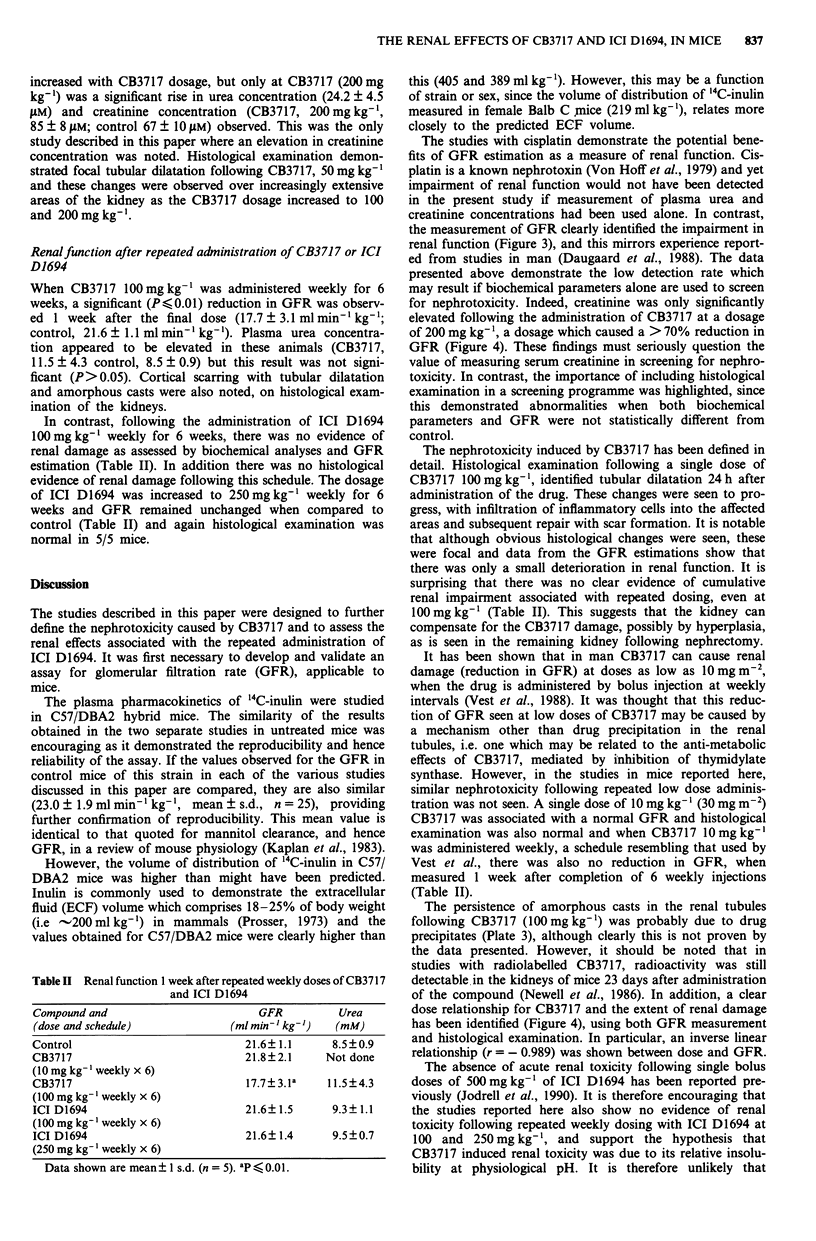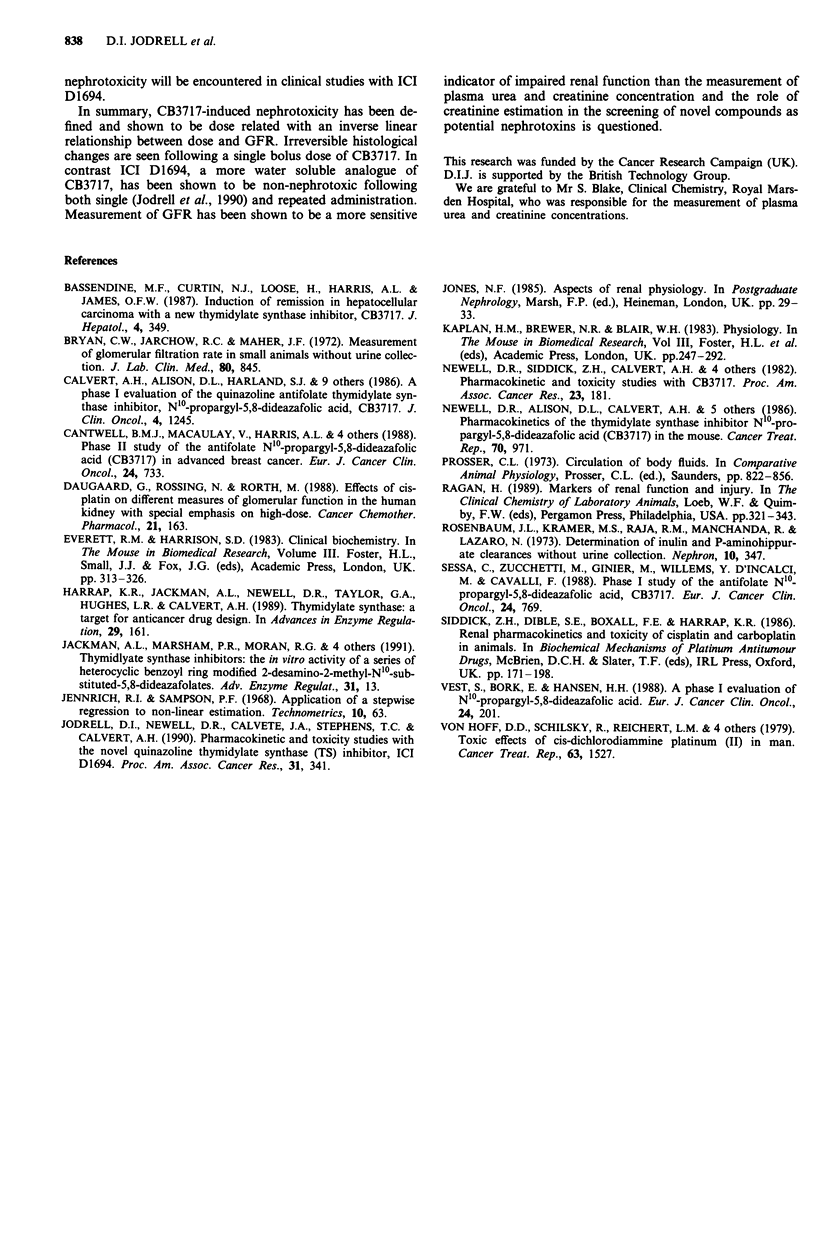# The renal effects of N10-propargyl-5,8-dideazafolic acid (CB3717) and a non-nephrotoxic analogue ICI D1694, in mice.

**DOI:** 10.1038/bjc.1991.409

**Published:** 1991-11

**Authors:** D. I. Jodrell, D. R. Newell, S. E. Morgan, S. Clinton, J. P. Bensted, L. R. Hughes, A. H. Calvert

**Affiliations:** Clinical Pharmacology Team, Drug Development Section, Sutton, Surrey, UK.

## Abstract

**Images:**


*Br. J. Cancer* (1991), **64**, 833–838 © Macmillan Press Ltd., 1991

### The renal effects of $N^{10}$-propargyl-5,8-dideazafolic acid (CB3717) and a non-nephrotoxic analogue ICI D1694, in mice

D.I. Jodrell[1], D.R. Newell[1,*], S.E. Morgan[1], S. Clinton[2], J.P.M. Bensted[2], L.R. Hughes[3] & A.H. Calvert[1,*]

[1]*Clinical Pharmacology Team, Drug Development Section, and* [2]*Histopathology Section, Institute of Cancer Research, 15 Cotswold Road, Sutton, Surrey SM2 5NG; and* [3]*ICI Pharmaceuticals, Mereside, Alderley Park, Macclesfield, Cheshire SK10 4TG, UK.*

**Summary** N-(5-[N-(3,4-dihydro-2-methyl-4-oxoquinazolin-6-ylmethyl)-N-methylamino]-2-thenoyl)-L-glutamic acid (ICI D1694) is an analogue of the thymidylate synthase inhibitor, $N^{10}$-propargyl-5,8-dideazafolic acid (CB3717). CB3717 was found to be active in early clinical studies, but its use was limited by nephrotoxicity. ICI D1694 is a more potent antitumour agent than CB3717 and is also more water soluble. Previous studies have shown ICI D1694 to be non-toxic to the kidney following a single administration but its renal effects after chronic administration are unknown. To assess these effects, and further define the time course and dose relationship of CB3717-induced renal damage, an assay of glomerular filtration rate (GFR) has been developed which can be used in mice and hence in the screening of novel compounds. The $^{14}$C-inulin clearance assay developed was used to show a linear relationship between CB3717 dosage and renal damage ($r = -0.989$) following a single bolus dose (50–200 mg kg$^{-1}$), in mice. CB3717-induced renal damage is persistent (>6 weeks) and renal scarring was noted. ICI D1694 has been shown to be non-nephrotoxic following weekly administration of 250 mg kg$^{-1}$ for 6 weeks. Measurement of GFR has been shown to be a more sensitive indicator of impaired renal function than plasma urea and creatinine concentration, and the measurement of plasma creatinine concentration in particular, appears to be without value in the screening of potential nephrotoxins in certain mouse strains.

$N^{10}$-propargyl-5,8-dideazafolic acid (CB3717, Figure 1.i.) is a folate based inhibitor of the enzyme thymidylate synthase (TS), which was found to be an active antitumour agent in early clinical trials in patients with breast, ovarian and hepatocellular carcinoma (Calvert *et al.*, 1986; Cantwell *et al.*, 1988; Bassendine *et al.*, 1987). However, the clinical use of CB3717 was limited by its nephrotoxicity, which was observed in Phase I studies of both weekly (Vest *et al.*, 1988) and 3-weekly administration schedules (Calvert *et al.*, 1986; Sessa *et al.*, 1988). Renal toxicity manifested primarily as a reduction in glomerular filtration rate (GFR). However, tubular damage was also identified by the measurement of the urinary enzymes, N-acetyl glucosaminidase (NAG) and leucine aminopeptidase (LAP). A >20% reduction in GFR was observed in seven of 12 (58%) patients receiving more than 400 mg m$^{-2}$ CB3717. In addition, urinary NAG and LAP levels were elevated in 50% of patients studied, although this elevation was not dose related (Calvert *et al.*, 1986).

CB3717-induced nephrotoxicity was thought to be due to the compound's relative insolubility at acid pH. However, despite adequate alkalinisation (pH ≥ 8) of the urine, reductions in GFR (measured using creatinine clearance) of >20%, were still seen in 6/17 (35%) courses in patients treated at 400 mg m$^{-2}$ (Sessa *et al.*, 1988). To circumvent toxicity related to drug precipitation in the kidney, a series of more soluble analogues of CB3717 have been synthesised. In addition to being devoid of renal toxicity in acute testing, a number of these compounds are more potent cytotoxic agents *in vitro* and retain antitumour activity *in vivo* (Harrap *et al.*, 1989). One such analogue, N-(5-[N-(3,4-dihydro-2-methyl- 4 -oxoquinazolin- 6 -ylmethyl)- N -methylamino]-2-thenoyl) L-glutamic acid (ICI D1694, Figure 1ii) has been selected for preclinical development (Jackman *et al.*, 1991).

The plasma concentrations of urea and creatinine are commonly measured to assess renal function during toxicological screening of new drugs. However, these parameters are relatively insensitive, because, in mice, a rise in plasma urea and creatinine concentration only occurs following the loss of 70–75% of functional renal mass (Everett & Harrison, 1983). Specificity is also poor because an elevation in plasma urea may be caused by 'pre-renal' (increased protein catabolism, gastric or intestinal bleeding and dehydration) or 'post-renal' (obstruction to renal outflow) causes, as well as parenchymal renal damage (Everett & Harrison, 1983). In patients treated with high dose cisplatin, attention has been drawn to the potential underestimation of the degree of renal damage by assessing renal function using serum creatinine levels alone (Daugaard *et al.*, 1988). In man, the measurement of GFR has become accepted as a routine method for the assessment of renal function. The classical technique using inulin, which is solely cleared from the plasma by glomerular filtration, involves its constant infusion with simultaneous urine collection (Jones, 1985). However, in mice, a constant infusion of inulin and simultaneous urine collection would require anaesthesia or rigid restraint and complete emptying of the bladder before and at completion of the test (Ragan, 1989). Therefore

**Figure 1** The structures of (i) CB3717 and (ii) ICI D1694.

Correspondence: D.I. Jodrell, Clinical Pharmacology, Drug Development Section, E Block, Institute of Cancer Research, 15 Cotswold Road, Sutton, Surrey SM2 5NG, UK.

*Present address: Cancer Research Unit, The Medical School, University of Newcastle upon Tyne, Framlington Place, Newcastle upon Tyne NE2 4HH, UK.

Received 5 June 1991; and in revised form 19 July 1991.

it is clearly unsuitable for routine toxicology screening. To simplify the measurement of GFR, the use of a single bolus injection, and the measurement of inulin clearance using a limited number of samples, has been studied and shown to correlate well with the classical method (Rosenbaum *et al.*, 1973). It is also possible to measure GFR using a single timed sample provided the volume of distribution (V) of the tracer is known (Bryan *et al.*, 1972). Renal clearance is calculated using the equation: Clearance = V ($\log_e(P_0/P_t)/t$), where t = time of sample, $P_0$ and $P_t$ = Plasma tracer concentration at $t_0$ and t. It is necessary to ascertain the volume of distribution for the tracer in the particular strain and sex of the species used for screening. It is then possible to assess the GFR of an individual animal from the dose administered and the plasma concentration at a single time point t, ($P_t$), because $P_0$ = Dose/V (provided t is in the elimination phase of tracer clearance).

In order to define the nephrotoxicity of CB3717 and assess the effects on the kidney following the repeat administration of ICI D1694 an assay using $^{14}C$ labelled inulin and a single timed plasma sample was developed and validated. Since cisplatin is a well documented nephrotoxin (Von Hoff *et al.*, 1979) it was used to validate the assay system and the results obtained were compared with plasma urea and creatinine concentrations, the classical markers for nephrotoxicity. Following validation of the assay, studies were performed to assess the renal impairment associated with both single and repeated administration of CB3717. Finally studies were performed to assess the effects on the kidney of repeated administration of the novel TS inhibitor chosen for clinical development, ICI D1694.

### Materials and methods

### *The measurement of glomerular filtration rate (GFR) in mice*

In order to define the volume of distribution of $^{14}C$-inulin, in female Balb C and in male C57/DBA2 F1 hybrid mice, $^{14}C$-inulin pharmacokinetics were studied in normal mice. Balb C mice are routinely used in the testing of platinum antitumour agents and C57/DBA2 mice in the testing of quinazoline TS inhibitors at the Institute of Cancer Research. $^{14}$Carbon labelled inulin (Amersham International PLC, Amersham, UK) with a specific activity of 2–10 mCi $mmol^{-1}$, was dissolved in PBS (0.01 M $Na_2PO_4$ pH 7.48) to give a 5 $\mu Ci\ ml^{-1}$ injection solution. Mice were briefly warmed to ⩽40°C and $^{14}C$-inulin was administered intravenously (iv) (0.01 ml $g^{-1}$ = 0.05 $\mu Ci\ g^{-1}$ body weight), via a tail vein at time $t_0$. Five mice were killed at time points predicted to fall in the elimination phase of inulin clearance i.e. 30, 40, 50 and 60 min. Following $CO_2$ asphyxiation, blood was collected by open cardiac puncture in a heparinised syringe. 0.1 ml of separated plasma was added to 1.0 ml of mixed quaternary ammonium hydroxides in toluene (NCS solubiliser, Amersham Corp., Illinois, USA) in a glass scintillation vial and incubated (40°C) overnight. NCS was neutralised by 0.04 ml acetic acid (17.45 M). Ten ml of scintillant (Emulsifier Safe, Hewlett Packard, Downers Grove, IL, USA) was added and radioactivity was measured by liquid scintillation (Hewlett Packard TRI-CARB Counter, 2000CA). Data obtained were then analysed by non-linear least squares regression analysis (Jennrich & Samson, 1968), using a weighting function of $1/(y + \hat{y})^2$. A monoexponential equation ($C = Ae^{-\alpha t}$) was fit to the data giving A (the plasma concentration at $t_0 = P_0$) and α (the rate constant). A was used to calculate the volume of distribution (V) using A = Dose/V. Inulin clearance and hence GFR was calculated using; Clearance = Vα.

In drug treated mice, 0.05 $\mu Ci\ g^{-1}$ body weight of $^{14}C$-inulin was administered iv at $t_0$ and plasma $^{14}C$-inulin concentration measured at 60 min (t), a time point shown to be in the elimination phase of $^{14}C$-inulin clearance (see results). The GFR in individual animals was calculated using Clearance = V ($\log_e(P_0/P_t)/t$). Group results are expressed as the mean ± 1 s.d. and compared using the Student's *t*-test. A comparison giving $P < 0.05$ was considered to be a significant difference.

### *Validation of the GFR assay using cisplatin*

Cisplatin (kindly provided by Johnson Matthew Research Group, Sonning, UK) was dissolved in 0.9% NaCl. Cisplatin 8 mg $kg^{-1}$, a dosage known to be nephrotoxic (Siddick *et al.*, 1986), was administered by intraperitoneal (ip) injection to Balb C mice. GFR was measured (as above) 4 days after cisplatin administration. Plasma urea and creatinine concentrations were measured using a COBAS BIO autoanalyser (Roche Diagnostics, Welwyn Garden City, UK) and commercially available kits (Boehringer Mannheim (urea) and Roche Diagnostics (creatinine)). These results were compared with those obtained from age matched untreated controls.

### *Investigation of CB3717-induced nephrotoxicity, following a single dose*

CB3717 (10 mg $ml^{-1}$ as disodium salt in $NaHCO_3$, ICI Pharmaceuticals) was administered ip to groups ($n = 5$) of C57/DBA2 mice at 100 mg $kg^{-1}$, a dosage known to be nephrotoxic (Newell *et al.*, 1982). Renal function was assessed 1, 3, 5, 7, 14, 20 and 42 days after the administration of CB3717.

To establish the relationship between CB3717 dose and renal toxicity, a range of CB3717 dosages (10, 50, 100 and 200 mg $kg^{-1}$) were administered as a single ip bolus. GFR was measured 5 days (chosen following time course experiment) after the administration of CB3717. Plasma urea and creatinine concentrations were also measured (see above). One kidney was removed and fixed (modified methacarn; 60% methanol, 30% Inhibisol (Kalon PLC, Cramlington, UK) and 10% acetic acid) prior to sectioning, staining (haematoxylin and eosin) and histological review, which was performed without prior knowledge of treatment details.

### *Renal function following repeated administration of CB3717 and ICI D1694*

CB3717 or ICI D1694 were administered by ip injection. ICI D1694 was supplied as a yellow powder and dissolved in 0.05 M $NaHCO_3$. pH was adjusted to 9.0–9.5 using NaOH. CB3717 (10 and 100 mg $kg^{-1}$) was administered weekly for 6 weeks and renal function assessed 1 week after the final dose. ICI D1694 was similarly administered at 100 and 250 mg $kg^{-1}$ $week^{-1}$. Renal function was assessed by GFR estimation, plasma urea and creatinine concentrations. Kidney tissue was prepared and examined histologically, as described above.

### Results

### *The plasma pharmacokinetics of $^{14}C$-inulin*

The plasma clearance of $^{14}C$-inulin, in both C57/DBA2 and Balb C mice, was fit by a single exponential decay (Figure 2). The volume of distribution of inulin in male C57/DBA2 F1 hybrid mice was calculated to be 405 and 389 ml $kg^{-1}$ body weight, in two separate studies. When $^{14}C$-inulin concentrations calculated by regression analysis were compared with the observed concentrations, to estimate the precision of the fit, linear regression (*r*) values of 0.986 and 0.951 were obtained, indicating that the data were well described by a single exponential term. The $^{14}C$-inulin clearance values, and hence GFR, were 21.9 and 20.6 ml $min^{-1}$ $kg^{-1}$ body weight in the two studies. In female Balb C mice the volume of distribution, 219 ml $kg^{-1}$, was lower than in male C57/DBA2 hybrid mice and the GFR was also lower at 15.3 ml $min^{-1}$ $kg^{-1}$.

### *Validation of the GFR assay using cisplatin*

Four days after the administration of cisplatin (8 mg $kg^{-1}$), a clear reduction in GFR was noted (Figure 3). This highly

**Figure 2** $^{14}$C-inulin clearance in female Balb C (--O--, 15.3 ml min$^{-1}$ kg$^{-1}$) and male C57/DBA2 hybrid (— + —, 20.6 ml min$^{-1}$ kg$^{-1}$) mice, following bolus intravenous injection.

significant ($P \leq 0.01$) reduction in GFR (control, 14.7 ± 0.8 ml min$^{-1}$ kg$^{-1}$; cisplatin 8 mg kg$^{-1}$, 6.7 ± 1.6 ml min$^{-1}$ kg$^{-1}$) was not associated with any significant rise in plasma urea and creatinine concentrations (Figure 3).

*Nephrotoxicity of CB3717 after a single administration*

GFR, plasma urea and creatinine concentrations and histological changes in the kidney were assessed up to 42 days after the administration of CB3717 (100 mg kg$^{-1}$) (Table I). A significant reduction in GFR was seen on day 3 and this persisted, with one exception (day 14), in all assays performed up to day 42. Plasma urea concentration was elevated on days 3 to 14, but plasma.creatinine concentration was not elevated at any stage. Histological examination was also found to be a sensitive marker of renal damage and histopathological damage evolved over the period of study. By day 3, focal tubular dilatation was clearly visible (Plate 2) when compared to the relatively normal histological appearance following a low dose of CB3717 (10 mg kg$^{-1}$) shown in Plate 1. Amorphous casts, which were thought to be drug precipitates, were noted within the tubular lumina. These casts persisted and were noted in sections, 20 days after administration of CB3717 (Plate 3) and were still present at 6 weeks. Tubular dilatation persisted through days 5 and 7 with increased tubular mitotic activity, suggesting that repair processes were commencing. By day 7 irregularities in the cortex due to contraction of scar tissue were becoming apparent. This contaction continued, and on day 20 depressions in the cortex and glomerular atrophy were seen (Plate 4). Six weeks after administration of CB3717 deeply pitted scars were clearly visible (Plate 5).

In addition plasma urea and creatinine concentrations were assayed and histological examinations were performed at 9, 10, 11 and 14 weeks in a small number of animals. Plasma biochemistry was normal in all these animals, however histological changes, such as cortical scarring and the presence of amorphous casts, were still visible at 14 weeks.

When renal function was assessed 5 days after CB3717 administered at increasing dosages, a clear inverse linear relationship ($r = -0.989$) between the CB3717 dosage and the degree of renal damage was observed (Figure 4). At 50 mg kg$^{-1}$, the reduction in GFR was significant (CB3717 50 mg kg$^{-1}$, 19.6 ± 1.2 ml min$^{-1}$ kg$^{-1}$; control, 21.9 ± 1.5 ml min$^{-1}$ kg$^{-1}$; $0.01 < P < 0.05$), but plasma urea concentration was not significantly elevated (CB3717 50 mg kg$^{-1}$, 6.4 ± 0.6 μM; control 8.8 ± 1.5 μM). The reduction in GFR

**Figure 3** Renal function assessed 4 days after cisplatin (8 mg kg$^{-1}$ ip). Data shown are mean ± 1 s.d. ($n = 5$).

**Table I** Renal function up to 42 days after 100 mg kg$^{-1}$ CB3717 as a single bolus

| *Time post injection (days)* | *GFR (ml min$^{-1}$ kg$^{-1}$)* | *Urea (mM)* | *Creatinine (μM)* |
|---|---|---|---|
| Control | 23.1±2.0 | 10.1±1.0 | 72±19 |
| 1 | 24.4±2.3 | 9.1±1.3 | 64±14 |
| 3 | 16.7±3.9[b] | 17.4±4.4[a] | 72±10 |
| 5 | 18.1±3.4[b] | 16.6±6.0[b] | 67± 9 |
| 7 | 17.7±3.6[b] | 13.4±3.3[a] | 61± 7 |
| 14 | 23.9±2.0 | 13.2±2.2[a] | 57± 3 |
| 20 | 18.9±2.0[b] | 10.4±1.2 | 50± 4 |
| 42 | 19.6±1.6[b] | 15.0±3.3 | 50± 3 |

Data shown are mean ± 1 s.d. ($n = 5$). [a]$0.01 < P < 0.05$; [b]$P \leq 0.01$.

**Plate 1** ( × 60 H&E) Relatively normal histological appearance of the kidney 5 days after a single low dose of CB3717 (10 mg $kg^{-1}$).

**Plate 2** ( × 60 H&E) Kidney section, 3 days after a single dose of CB3717 (100 mg $kg^{-1}$), showing tubular dilatation.

**Plate 3** ( × 240 H&E) Kidney section to show the tubular casts persisting 20 days after a single dose of CB3717 (100 mg $kg^{-1}$).

**Plate 4** ( × 60 H&E) Early, mild cortical scarring with glomerular atrophy in the scars, 20 days after a single dose of CB3717 (100 mg $kg^{-1}$).

**Plate 5** ( × 60 H&E) Deep cortical scars, 42 days after a single dose of CB3717 (100 mg $kg^{-1}$).

**Figure 4** The apparent linear relationship ($r = -0.989$) between glomerular filtration rate (GFR) and CB3717 dosage administered 5 days prior to GFR estimation. Data shown are mean ± 1 s.d. ($n = 5$).

increased with CB3717 dosage, but only at CB3717 (200 mg $kg^{-1}$) was a significant rise in urea concentration (24.2 ± 4.5 μM) and creatinine concentration (CB3717, 200 mg $kg^{-1}$, 85 ± 8 μM; control 67 ± 10 μM) observed. This was the only study described in this paper where an elevation in creatinine concentration was noted. Histological examination demonstrated focal tubular dilatation following CB3717, 50 mg $kg^{-1}$ and these changes were observed over increasingly extensive areas of the kidney as the CB3717 dosage increased to 100 and 200 mg $kg^{-1}$.

*Renal function after repeated administration of CB3717 or ICI D1694*

When CB3717 100 mg $kg^{-1}$ was administered weekly for 6 weeks, a significant ($P \leqslant 0.01$) reduction in GFR was observed 1 week after the final dose (17.7 ± 3.1 ml $min^{-1}$ $kg^{-1}$; control, 21.6 ± 1.1 ml $min^{-1}$ $kg^{-1}$). Plasma urea concentration appeared to be elevated in these animals (CB3717, 11.5 ± 4.3 control, 8.5 ± 0.9) but this result was not significant ($P > 0.05$). Cortical scarring with tubular dilatation and amorphous casts were also noted, on histological examination of the kidneys.

In contrast, following the administration of ICI D1694 100 mg $kg^{-1}$ weekly for 6 weeks, there was no evidence of renal damage as assessed by biochemical analyses and GFR estimation (Table II). In addition there was no histological evidence of renal damage following this schedule. The dosage of ICI D1694 was increased to 250 mg $kg^{-1}$ weekly for 6 weeks and GFR remained unchanged when compared to control (Table II) and again histological examination was normal in 5/5 mice.

### Discussion

The studies described in this paper were designed to further define the nephrotoxicity caused by CB3717 and to assess the renal effects associated with the repeated administration of ICI D1694. It was first necessary to develop and validate an assay for glomerular filtration rate (GFR), applicable to mice.

The plasma pharmacokinetics of $^{14}$C-inulin were studied in C57/DBA2 hybrid mice. The similarity of the results obtained in the two separate studies in untreated mice was encouraging as it demonstrated the reproducibility and hence reliability of the assay. If the values observed for the GFR in control mice of this strain in each of the various studies discussed in this paper are compared, they are also similar (23.0 ± 1.9 ml $min^{-1}$ $kg^{-1}$, mean ± s.d., $n = 25$), providing further confirmation of reproducibility. This mean value is identical to that quoted for mannitol clearance, and hence GFR, in a review of mouse physiology (Kaplan *et al.*, 1983).

However, the volume of distribution of $^{14}$C-inulin in C57/DBA2 mice was higher than might have been predicted. Inulin is commonly used to demonstrate the extracellular fluid (ECF) volume which comprises 18–25% of body weight (i.e ~200 ml $kg^{-1}$) in mammals (Prosser, 1973) and the values obtained for C57/DBA2 mice were clearly higher than this (405 and 389 ml $kg^{-1}$). However, this may be a function of strain or sex, since the volume of distribution of $^{14}$C-inulin measured in female Balb C mice (219 ml $kg^{-1}$), relates more closely to the predicted ECF volume.

The studies with cisplatin demonstrate the potential benefits of GFR estimation as a measure of renal function. Cisplatin is a known nephrotoxin (Von Hoff *et al.*, 1979) and yet impairment of renal function would not have been detected in the present study if measurement of plasma urea and creatinine concentrations had been used alone. In contrast, the measurement of GFR clearly identified the impairment in renal function (Figure 3), and this mirrors experience reported from studies in man (Daugaard *et al.*, 1988). The data presented above demonstrate the low detection rate which may result if biochemical parameters alone are used to screen for nephrotoxicity. Indeed, creatinine was only significantly elevated following the administration of CB3717 at a dosage of 200 mg $kg^{-1}$, a dosage which caused a > 70% reduction in GFR (Figure 4). These findings must seriously question the value of measuring serum creatinine in screening for nephrotoxicity. In contrast, the importance of including histological examination in a screening programme was highlighted, since this demonstrated abnormalities when both biochemical parameters and GFR were not statistically different from control.

The nephrotoxicity induced by CB3717 has been defined in detail. Histological examination following a single dose of CB3717 100 mg $kg^{-1}$, identified tubular dilatation 24 h after administration of the drug. These changes were seen to progress, with infiltration of inflammatory cells into the affected areas and subsequent repair with scar formation. It is notable that although obvious histological changes were seen, these were focal and data from the GFR estimations show that there was only a small deterioration in renal function. It is surprising that there was no clear evidence of cumulative renal impairment associated with repeated dosing, even at 100 mg $kg^{-1}$ (Table II). This suggests that the kidney can compensate for the CB3717 damage, possibly by hyperplasia, as is seen in the remaining kidney following nephrectomy.

It has been shown that in man CB3717 can cause renal damage (reduction in GFR) at doses as low as 10 mg $m^{-2}$, when the drug is administered by bolus injection at weekly intervals (Vest *et al.*, 1988). It was thought that this reduction of GFR seen at low doses of CB3717 may be caused by a mechanism other than drug precipitation in the renal tubules, i.e. one which may be related to the anti-metabolic effects of CB3717, mediated by inhibition of thymidylate synthase. However, in the studies in mice reported here, similar nephrotoxicity following repeated low dose administration was not seen. A single dose of 10 mg $kg^{-1}$ (30 mg $m^{-2}$) CB3717 was associated with a normal GFR and histological examination was also normal and when CB3717 10 mg $kg^{-1}$ was administered weekly, a schedule resembling that used by Vest *et al.*, there was also no reduction in GFR, when measured 1 week after completion of 6 weekly injections (Table II).

The persistence of amorphous casts in the renal tubules following CB3717 (100 mg $kg^{-1}$) was probably due to drug precipitates (Plate 3), although clearly this is not proven by the data presented. However, it should be noted that in studies with radiolabelled CB3717, radioactivity was still detectable in the kidneys of mice 23 days after administration of the compound (Newell *et al.*, 1986). In addition, a clear dose relationship for CB3717 and the extent of renal damage has been identified (Figure 4), using both GFR measurement and histological examination. In particular, an inverse linear relationship ($r = -0.989$) was shown between dose and GFR.

The absence of acute renal toxicity following single bolus doses of 500 mg $kg^{-1}$ of ICI D1694 has been reported previously (Jodrell *et al.*, 1990). It is therefore encouraging that the studies reported here also show no evidence of renal toxicity following repeated weekly dosing with ICI D1694 at 100 and 250 mg $kg^{-1}$, and support the hypothesis that CB3717 induced renal toxicity was due to its relative insolubility at physiological pH. It is therefore unlikely that

**Table II** Renal function 1 week after repeated weekly doses of CB3717 and ICI D1694

| *Compound and (dose and schedule)* | *GFR ($ml\ min^{-1}\ kg^{-1}$)* | *Urea (mM)* |
|---|---|---|
| Control | 21.6±1.1 | 8.5±0.9 |
| CB3717 (10 mg $kg^{-1}$ weekly × 6) | 21.8±2.1 | Not done |
| CB3717 (100 mg $kg^{-1}$ weekly × 6) | 17.7±3.1[a] | 11.5±4.3 |
| ICI D1694 (100 mg $kg^{-1}$ weekly × 6) | 21.6±1.5 | 9.3±1.1 |
| ICI D1694 (250 mg $kg^{-1}$ weekly × 6) | 21.6±1.4 | 9.5±0.7 |

Data shown are mean ± 1 s.d. ($n = 5$). [a]$P \leqslant 0.01$.

nephrotoxicity will be encountered in clinical studies with ICI D1694.

In summary, CB3717-induced nephrotoxicity has been defined and shown to be dose related with an inverse linear relationship between dose and GFR. Irreversible histological changes are seen following a single bolus dose of CB3717. In contrast ICI D1694, a more water soluble analogue of CB3717, has been shown to be non-nephrotoxic following both single (Jodrell *et al.*, 1990) and repeated administration. Measurement of GFR has been shown to be a more sensitive indicator of impaired renal function than the measurement of plasma urea and creatinine concentration and the role of creatinine estimation in the screening of novel compounds as potential nephrotoxins is questioned.

This research was funded by the Cancer Research Campaign (UK). D.I.J. is supported by the British Technology Group.

We are grateful to Mr S. Blake, Clinical Chemistry, Royal Marsden Hospital, who was responsible for the measurement of plasma urea and creatinine concentrations.

### References

BASSENDINE, M.F., CURTIN, N.J., LOOSE, H., HARRIS, A.L. & JAMES, O.F.W. (1987). Induction of remission in hepatocellular carcinoma with a new thymidylate synthase inhibitor, CB3717. *J. Hepatol.*, **4**, 349.

BRYAN, C.W., JARCHOW, R.C. & MAHER, J.F. (1972). Measurement of glomerular filtration rate in small animals without urine collection. *J. Lab. Clin. Med.*, **80**, 845.

CALVERT, A.H., ALISON, D.L., HARLAND, S.J. & 9 others (1986). A phase I evaluation of the quinazoline antifolate thymidylate synthase inhibitor, $N^{10}$-propargyl-5,8-dideazafolic acid, CB3717. *J. Clin. Oncol.*, **4**, 1245.

CANTWELL, B.M.J., MACAULAY, V., HARRIS, A.L. & 4 others (1988). Phase II study of the antifolate $N^{10}$-propargyl-5,8-dideazafolic acid (CB3717) in advanced breast cancer. *Eur. J. Cancer Clin. Oncol.*, **24**, 733.

DAUGAARD, G., ROSSING, N. & RORTH, M. (1988). Effects of cisplatin on different measures of glomerular function in the human kidney with special emphasis on high-dose. *Cancer Chemother. Pharmacol.*, **21**, 163.

EVERETT, R.M. & HARRISON, S.D. (1983). Clinical biochemistry. In *The Mouse in Biomedical Research*, Volume III. Foster, H.L., Small, J.J. & Fox, J.G. (eds), Academic Press, London, UK. pp. 313–326.

HARRAP, K.R., JACKMAN, A.L., NEWELL, D.R., TAYLOR, G.A., HUGHES, L.R. & CALVERT, A.H. (1989). Thymidylate synthase: a target for anticancer drug design. In *Advances in Enzyme Regulation*, **29**, 161.

JACKMAN, A.L., MARSHAM, P.R., MORAN, R.G. & 4 others (1991). Thymidlyate synthase inhibitors: the *in vitro* activity of a series of heterocyclic benzoyl ring modified 2-desamino-2-methyl-$N^{10}$-substituted-5,8-dideazafolates. *Adv. Enzyme Regulat.*, **31**, 13.

JENNRICH, R.I. & SAMPSON, P.F. (1968). Application of a stepwise regression to non-linear estimation. *Technometrics*, **10**, 63.

JODRELL, D.I., NEWELL, D.R., CALVETE, J.A., STEPHENS, T.C. & CALVERT, A.H. (1990). Pharmacokinetic and toxicity studies with the novel quinazoline thymidylate synthase (TS) inhibitor, ICI D1694. *Proc. Am. Assoc. Cancer Res.*, **31**, 341.

JONES, N.F. (1985). Aspects of renal physiology. In *Postgraduate Nephrology*, Marsh, F.P. (ed.), Heineman, London, UK. pp. 29–33.

KAPLAN, H.M., BREWER, N.R. & BLAIR, W.H. (1983). Physiology. In *The Mouse in Biomedical Research*, Vol III, Foster, H.L. *et al.* (eds), Academic Press, London, UK. pp.247–292.

NEWELL, D.R., SIDDICK, Z.H., CALVERT, A.H. & 4 others (1982). Pharmacokinetic and toxicity studies with CB3717. *Proc. Am. Assoc. Cancer Res.*, **23**, 181.

NEWELL, D.R., ALISON, D.L., CALVERT, A.H. & 5 others (1986). Pharmacokinetics of the thymidylate synthase inhibitor $N^{10}$-propargyl-5,8-dideazafolic acid (CB3717) in the mouse. *Cancer Treat. Rep.*, **70**, 971.

PROSSER, C.L. (1973). Circulation of body fluids. In *Comparative Animal Physiology*, Prosser, C.L. (ed.), Saunders, pp. 822–856.

RAGAN, H. (1989). Markers of renal function and injury. In *The Clinical Chemistry of Laboratory Animals*, Loeb, W.F. & Quimby, F.W. (eds), Pergamon Press, Philadelphia, USA. pp.321–343.

ROSENBAUM, J.L., KRAMER, M.S., RAJA, R.M., MANCHANDA, R. & LAZARO, N. (1973). Determination of inulin and P-aminohippurate clearances without urine collection. *Nephron*, **10**, 347.

SESSA, C., ZUCCHETTI, M., GINIER, M., WILLEMS, Y. D'INCALCI, M. & CAVALLI, F. (1988). Phase I study of the antifolate $N^{10}$-propargyl-5,8-dideazafolic acid, CB3717. *Eur. J. Cancer Clin. Oncol.*, **24**, 769.

SIDDICK, Z.H., DIBLE, S.E., BOXALL, F.E. & HARRAP, K.R. (1986). Renal pharmacokinetics and toxicity of cisplatin and carboplatin in animals. In *Biochemical Mechanisms of Platinum Antitumour Drugs*, McBrien, D.C.H. & Slater, T.F. (eds), IRL Press, Oxford, UK. pp. 171–198.

VEST, S., BORK, E. & HANSEN, H.H. (1988). A phase I evaluation of $N^{10}$-propargyl-5,8-dideazafolic acid. *Eur. J. Cancer Clin. Oncol.*, **24**, 201.

VON HOFF, D.D., SCHILSKY, R., REICHERT, L.M. & 4 others (1979). Toxic effects of cis-dichlorodiammine platinum (II) in man. *Cancer Treat. Rep.*, **63**, 1527.